# Pediatric cardiac patients with pulmonary hemorrhage supported on ECMO: An ELSO registry study

**DOI:** 10.1051/ject/2024038

**Published:** 2025-03-07

**Authors:** Pilar Anton-Martin, Caroline Young, Hitesh Sandhu, Shilpa Vellore

**Affiliations:** 1 Department of Pediatrics, Division of Anesthesiology and Critical Care Medicine, Children’s Hospital of Philadelphia Philadelphia PA 19130 USA; 2 Department of Pediatrics, Division of Critical Care, Emory University School of Medicine/Children’s Healthcare of Atlanta Atlanta GA 30329 USA; 3 Department of Pediatrics, Division of Critical Care, University of Tennessee Health Science Center/Le Bonheur Children’s Hospital Memphis TN 38103 USA; 4 Department of Pediatrics, Division of Cardiology, University of California San Diego School of Medicine/Rady Children’s Hospital San Diego CA 92123 USA

**Keywords:** ECMO, High-frequency oscillatory ventilation, Children, Heart disease, Survival

## Abstract

*Background:* Pulmonary Hemorrhage (PH) is a rare but potentially devastating condition and pediatric cardiac patients are at increased risk for. ECMO may be used to safely support these patients, but data is limited. *Methods:* Observational retrospective cohort study from the ELSO registry database in pediatric cardiac patients from birth to 18 years old with PH supported on ECMO from January 2011 through December 2020. The objectives of the study were to characterize pediatric cardiac patients with PH before ECMO and to describe factors associated with improved survival. *Results:* A total of 161 cardiac neonates and children with PH supported on ECMO were analyzed. Median age and weight were 40 days (IQR 7.3–452) and 4.06 kg (IQR 3–9.36), respectively. Congenital heart disease accounted for 77% of diagnoses. Survival to hospital discharge was 35.8%. Before ECMO cannulation, most patients were ventilated in conventional modes (79.7%), followed by high-frequency oscillatory (HFOV) ventilation (11%). There was a significantly higher use of HFOV pre-cannulation in survivors compared to non-survivors (24.4% vs 2.8%, *p* < 0.001). Multivariable logistic regression demonstrated that HFOV before ECMO (OR 28.44, *p* < 0.001) and the absence of hemorrhagic (OR 3.51, *p* 0.031) and renal (OR 3.50, *p* 0.027) complications were independent predictors for survival to hospital discharge. *Conclusion:* Utilization of HFOV before cannulation to ECMO seems to be associated with improved survival in pediatric cardiac patients with acute pulmonary hemorrhage. A prospective assessment of mechanical ventilation practices before ECMO may improve outcomes in this medically complex population.

## Introduction

Pulmonary Hemorrhage (PH) is a rare but potentially devastating condition in neonates and children. Massive bleeding may occur secondary to vascular injury (infections, immune-mediated processes like vasculitides, drug toxicity), abnormal architecture of the pulmonary vasculature (either congenital or acquired), trauma, etc. [1]. The incidence of massive PH in children is variable depending upon the cause and population reviewed [2]. In their 10-year review of the causes of hemoptysis at a single large institution, Coss Bu et al. reported that the most frequent causes were cystic fibrosis in 65%, congenital heart disease (CHD) in 16%, with the remaining 19% being due to infections, neoplasms, and other causes [2].

Patients with CHD are at increased risk of PH due to multiple factors [3, 4]. However, the true incidence of PH within this subpopulation is particularly difficult to determine due to the paucity of classic PH symptoms [3, 4]. While traditionally hemoptysis, pulmonary infiltrates, and anemia are seen in PH, one or multiple of these signs may be absent or not connected to a unified PH diagnosis in pediatric cardiac patients [3, 5].

Patients with severe PH have been successfully treated with high-frequency oscillatory ventilation (HFOV) [6]. Additionally, extracorporeal membrane oxygenation (ECMO) may be used safely to support pediatric patients with PH, however, outcome data is limited [7–13]. While HFOV may be utilized in pediatric heart disease patients with respiratory failure, its use is limited in this population due to the potential for worsening cardiopulmonary interactions [14]. Furthermore, the outcomes of pediatric patients with heart disease who received HFOV as a ventilatory strategy for PH before ECMO cannulation remain unclear. Our study aimed to evaluate predictors of survival to hospital discharge in pediatric cardiac patients with PH requiring ECMO.

## Materials and methods

### Study setting and design

This study was an observational, retrospective cohort study that utilized the Extracorporeal Life Support Organization (ELSO) registry database. The Institutional Review Board at the University of Tennessee Health Science Center reviewed the study and determined it to be Not Human Subjects Research status.

### Study population and data collection

**Figure 1:**
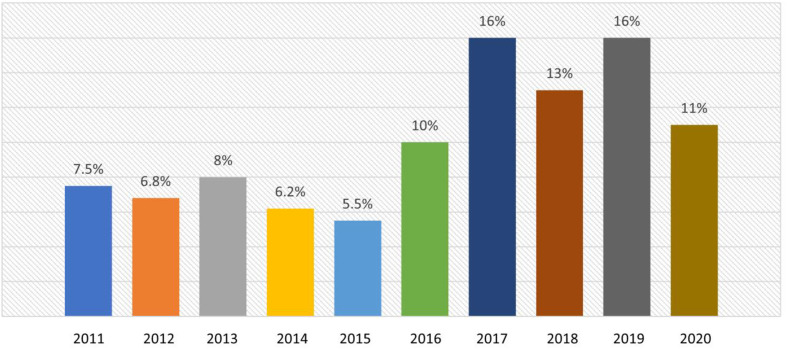
Distribution of cardiac patients with pulmonary hemorrhage supported on ECMO over time.

All neonatal and pediatric cardiac patients ≤18 years of age with PH supported on ECMO between January 1, 2011, and December 31, 2020, were included in this study. PH was identified using the International Classification of Diseases 9 and 10 codes utilized to identify secondary diagnoses in the ELSO database. ECMO runs with inaccurate data as well as secondary and subsequent runs were excluded. We also excluded those patients who developed PH as an ECMO complication. The data extracted from the ELSO registry database included information regarding demographics, cardiac diagnoses, use of cardiopulmonary bypass (CPB) before cannulation, pre-extracorporeal life support, ECMO support, complications, and outcomes. Cardiac diagnoses were dichotomized as CHD and heart failure (cardiomyopathy, myocarditis, heart transplant, etc.). Ventilator support was grouped as conventional ventilation, HFOV, and other ventilator modes. The severity of illness indicators available in the dataset at the time of ECMO initiation included pH, oxygenation index (OI), mean blood pressure, arrest before ECMO, nitric oxide use, and renal replacement therapy use. ECMO type was grouped into veno-venous and veno-arterial (VA) and ECMO indication was categorized into pulmonary, cardiac, and extracorporeal cardiopulmonary resuscitation (ECPR). Year of ECMO data was also available. However, since most of the patients were clustered over the last 5 years, we did not perform any analysis to evaluate the influence of temporal trends on the outcomes (Fig. 1).

### Aims, hypothesis, and outcomes

We aimed to characterize the population of neonatal and pediatric cardiac patients with PH supported on ECMO and to describe factors associated with improved survival. We hypothesized that these patients would benefit from HFOV before cannulation. The primary outcome was survival to hospital discharge. Secondary outcomes were ECMO duration, hospital length of stay (LOS), and mechanical ventilation (MV) duration.

### Statistical analysis

Continuous variables were described using medians and interquartile ranges (IQR) while frequencies and proportions were used for categorical variables. Bivariate analyses were conducted using Chi-squared tests and Wilcoxon-Mann-Whitney tests to ascertain the association between covariates and outcomes. Multivariable logistic regression models were used to analyze the effects of potential variables on survival to hospital discharge. Backward selection with an alpha level of removal of 0.05 was utilized. Odds ratios (OR) and 95% confidence intervals were calculated. All *p*-values were 2-sided and *p* < 0.05 was considered statistically significant. Statistical analyses were performed using SAS (version 9.4, SAS Institute Inc., NC, USA).

## Results

### Patient population

A total of 161 cardiac neonates and children with PH supported on ECMO between January 2011 and December 2020 were included in this study. The median age and weight of the cohort were 40 days (IQR 7.3–452) and 4.06 kg (IQR 3–9.36). Neonates (<30 days old) accounted for 48.1% of patients. Males were predominant (59%). CHD accounted for 77.2% of diagnoses. The most frequent cardiac diagnoses were transposition of the great arteries (17.3%), hypoplastic left heart syndrome (15.4%), double outlet right ventricle (10%), cardiomyopathy (8.6%), and heart transplant (6.2%). Survival to hospital discharge reached 36% (Table 1).

**Table 1:** Characteristics of survivors and non-survivors to hospital discharge.

Characteristics	All cohort (*n* = 161)	Survivors to hospital discharge (*n* = 58, 36%)	Non-survivors to hospital discharge (*n* = 103, 64%)	*p*-value
**Demographic data**				
**Age (years)**^a^	0.11 (0.02, 1.24)	0.1 (0, 0.9)	0.1 (0, 1.5)	0.238
**Age group**^b^				0.973
Neonate (≤30 days)	78 (48.1)	28 (48.3)	50 (48.5)	
Pediatric	83 (51.5)	30 (51.7)	53 (51.5)	
**Weight (kg)**^a^	4.06 (3, 9.36)	4.2 (3.3, 8.6)	4 (3, 10.4)	0.805
**Height (cm)**^a^	62.5 (49, 95.3)	59.5 (51, 73)	65.8 (49, 107.4)	0.491
**BSA (m**^**2**^**)**^a^	0.31 (0.21, 0.62)	0.3 (0.2, 0.5)	0.4 (0.2, 0.7)	0.661
**Gender**^b^				0.232
Female	63 (39.6)	19 (32.8)	44 (44)	
Male	95 (59)	39 (67.2)	56 (56)	
**Race**^b^				0.854
Caucasian	91 (56.5)	32 (55.2)	59 (57.3)	
African-American	27 (16.7)	11 (19)	16 (15.5)	
Other	43 (26.8)	15 (25.8)	28 (27.2)	
**Diagnostic group**^b^				0.055
CHD	125 (77.2)	49 (84.5)	76 (73.8)	
Heart failure	36 (22.3)	9 (15.5)	27 (26.2)	
**Heart transplant**^b^	10 (6.2)	3 (5.2)	7 (6.8)	0.681
**Pre-ECMO data and support**				
**Ventilator support**^b^				**<0.001**
Conventional	94 (79.7)	33 (73.3)	60 (83.3)	
HFOV	13 (11)	11 (24.4)	2 (2.8)	
Other	11 (6.8)	1 (2.2)	10 (13.9)	
**Pre ECMO arrest**^b^	63 (39)	17 (29.3)	46 (45.1)	**0.044**
**Oxygenation index**^a^	35 (14, 55)	39 (23, 57)	32.5 (10.5, 51)	0.154
**Mean BP (mmHg)**^a^	41.5 (32, 53)	42 (32, 53)	41 (31.5, 52.5)	0.918
**FiO2 need (%)**^a^	100 (87, 100)	100 (91, 100)	100 (82, 100)	0.507
**pH**^a^	7.2 (7.05, 7.32)	7.2 (7.1, 7.3)	7.2 (7, 7.3)	0.485
**CPB**^b^	63 (39)	27 (46.6)	36 (35)	0.232
**VAD**^b^	10 (6.2)	4 (6.9)	6 (5.8)	0.768
**RRT**^b^	2 (1.2)	0 (0)	2 (1.9)	0.540
**Nitric oxide**^b^	56 (34.7)	24 (41.4)	32 (31.1)	0.325
**ECMO data**				
**ECMO mode**^b^				0.843
Veno-arterial	153 (94.4)	55 (94.8)	97 (94.2)	
Veno-venous	7 (4.3)	2 (3.4)	5 (4.9)	
Unknown	2 (1.2)	1 (1.7)	1 (1)	
**ECMO type**^b^				0.269
Cardiac	114 (70.4)	42 (72.4)	72 (69.9)	
Pulmonary	10 (6.2)	5 (8.6)	5 (4.9)	
ECPR	38 (23.5)	11 (19)	26 (25.2)	
**Cannula location**^b^				0.396
Central	97 (60.6)	37 (63.8)	60 (59.4)	
Peripheral	63 (39.4)	21 (36.2)	41 (40.6)	
**Cardiac index**^a^	1.97 (1.57, 2.47)	2 (1.5, 2.4)	1.9 (1.6, 2.5)	0.853
**Outcomes and complications**				
**ECMO duration (h)**^a^	130.5 (73, 218)	105.5 (81, 161)	137 (53, 312)	0.222
**LOS (days)**^a^	29 (16.5, 53.5)	43.5 (29, 109)	23 (11, 36)	**<0.001**
**MV duration (h)**^a^	356 (172, 654)	387 (240, 721)	315 (125, 580)	**0.01**
**Complication type**^b^				
Cardiovascular	71 (43.8)	15 (25.9)	56 (54.4)	**<0.001**
Hemorrhagic	78 (48.1)	18 (31)	60 (58.3)	**0.001**
Infectious	12 (7.4)	1 (1.7)	11 (10.7)	0.127
Limb	4 (2.5)	0 (0)	4 (3.9)	0.322
Mechanical	53 (32.7)	11 (19)	42 (40.8)	**0.006**
Metabolic	41 (25.3)	8 (13.8)	33 (32)	**0.017**
Neurologic	35 (21.6)	6 (10.3)	29 (28.2)	**0.012**
Respiratory	44 (27.2)	6 (10.3)	38 (36.9)	**<0.001**
Renal	73 (45.1)	19 (32.8)	54 (52.4)	**0.023**

a Medians (IQR).

b Frequencies (%).

BP: blood pressure, BSA: body surface area, CHD: congenital heart disease, CPB: cardiopulmonary bypass, ECMO: extracorporeal membrane oxygenation, ECPR: extracorporeal cardiopulmonary resuscitation, FiO2: fraction of inspired oxygen, HFOV: high-frequency oscillatory ventilation, h: hours, LOS: length of stay, MV: mechanical ventilation, RRT: renal replacement therapy, VAD: ventricular assist device.

Heart failure includes cardiomyopathy, myocarditis, and heart transplant.

### Support before ECMO, characteristics, and complications

Before ECMO cannulation, most patients were ventilated in conventional modes (79.7%), followed by HFOV (11%) and other ventilator types (6.8%). VA support was the most frequent ECMO mode (94.4%) and cannulation via ECPR occurred in 23.5% of patients. The most frequent ECMO complications in the cohort included the need for renal replacement therapy (44%) and surgical site bleeding (25.3%). Table 1 summarizes the above data.

### Patients ventilated on conventional vs HFOV before ECMO

Patients ventilated on HFOV before ECMO had a higher OI (*p* < 0.001), were more often classified as pulmonary ECMO type (*p* < 0.001) and exhibited reduced utilization of CPB (*p* = 0.018) and central cannulation (*p* = 0.041). Table 2 provides a comparison of pre-ECMO support, characteristics, and complications between patients who received conventional ventilation vs HFOV prior to ECMO cannulation.

**Table 2:** Characteristics of patients that received conventional ventilation vs HFOV prior to ECMO cannulation.

Characteristics	Conventional (*n* = 94)	HFOV (*n* = 13)	*p*-value
**Demographic data**			
**Age (years)**^a^	0.12 (0.02, 1.02)	0.5 (0.01, 1.98)	0.942
**Age group**^b^			0.920
Neonate (≤30 days)	42 (44.5)	6 (46)	
Pediatric	52 (55.5)	7 (54)	
**Weight (kg)**^a^	4.2 (3, 9)	5.6 (3, 12)	0.432
**Height (cm)**^a^	64.5 (50, 98)	58.7 (48, 77)	0.327
**BSA (m**^**2**^**)**^a^	0.32 (0.22, 0.62)	0.28 (0.20, 0.47)	0.416
**Gender**^b^			0.368
Female	40 (42.5)	4 (30.7)	
Male	51 (57.5)	9 (69.3)	
**Race**^b^			0.349
Caucasian	49 (52)	4 (31)	
African-American	16 (17)	3 (22)	
Other	29 (31)	6 (47)	
**Diagnostic group**^b^			0.501
CHD	73 (77.6)	9 (69)	
Heart failure	21 (22.4)	4 (31)	
**Heart transplant**^b^	8 (8.5)	0 (0)	N/A
**Pre-ECMO data and support**			
**Pre ECMO arrest**^b^	33 (35)	4 (31)	0.757
**Oxygenation index**^a^	27 (12, 48)	43 (45, 88)	**<0.001**
**Mean BP (mmHg)**^a^	43 (33, 53)	62 (28, 58)	0.902
**FiO2 need (%)**^a^	100 (75, 100)	100 (100, 100)	0.092
**pH**^a^	7.19 (7.10, 7.31)	7.34 (7.05, 7.34)	0.819
**CPB**^b^	39 (41.4)	1 (7.6)	**0.018**
**VAD**^b^	9 (9.5)	0 (0)	N/A
**RRT**^b^	1 (1)	0 (0)	N/A
**Nitric oxide**^b^	41 (43.6)	6 (46)	0.862
**ECMO data**			
**ECMO mode**^b^			0.974
Veno-arterial	87 (92.5)	12 (92.3)	
Veno-Venous	7 (7.5)	1 (7.6)	
**ECMO type**^b^			**<0.001**
Cardiac	76 (80.8)	5 (38.4)	
Pulmonary	5 (5.3)	5 (38.4)	
ECPR	13 (13.9)	3 (23.2)	
**Cannula location**^b^			**0.041**
Central	57 (60.6)	4 (31)	
Peripheral	37 (39.4)	9 (69)	
**Cardiac index**^a^	1.97 (1.6, 2.5)	1.97 (1.5, 2.0)	0.482
**Outcomes and complications**			
**ECMO duration (h)**^a^	137 (90, 282)	130 (72, 163)	0.303
**LOS (days)**^a^	31 (21, 56)	38 (22, 53)	0.732
**MV duration (h)**^a^	378 (216, 742)	364 (201, 566)	0.660
**Complication type**^b^			
Cardiovascular	40 (42.5)	4 (31)	0.418
Hemorrhagic	39 (41.4)	4 (31)	0.459
Infectious	6 (6.3)	1 (7.6)	0.857
Limb	1 (1)	0 (0)	N/A
Mechanical	39 (41.4)	2 (15.4)	0.069
Metabolic	21 (22.3)	3 (23)	0.952
Neurologic	17 (18)	1 (7.6)	0.347
Respiratory	25 (26.5)	2 (15.4)	0.383
Renal	42 (44.6)	7 (53.8)	0.534

a Medians (IQR).

b Frequencies (%).

BP: blood pressure, BSA: body surface area, CHD: congenital heart disease, CPB: cardiopulmonary bypass, ECMO: extracorporeal membrane oxygenation, ECPR: extracorporeal cardiopulmonary resuscitation, FiO2: fraction of inspired oxygen, HFOV: high-frequency oscillatory ventilation, h: hours, LOS: length of stay, MV: mechanical ventilation, RRT: renal replacement therapy, VAD: ventricular assist device.

Heart failure includes cardiomyopathy, myocarditis, and heart transplant.

### Survivors and non-survivors to hospital discharge

No differences were observed in demographic and diagnostic characteristics between both groups. There was a significantly higher use of HFOV pre-cannulation in survivors compared to non-survivors (24.4% vs 2.8%, *p* < 0.001). Non-survivors significantly had more arrests before ECMO than survivors (45.1% vs 29.3%, *p* 0.044). No differences were observed in ECMO mode and type, cannulation location, and cardiac index provided for support. Survivors had significantly longer LOS and MV duration than non-survivors (43.5 vs 23 days, *p* < 0.001 and 387 vs 315 h, *p* 0.01; respectively) likely due to early deaths in the latter. Non-survivors had significantly more cardiovascular, hemorrhagic, mechanical, metabolic, neurologic, respiratory, and renal complications (Table 1).

### Factors associated with survival to hospital discharge

Multivariable logistic regression models were used to evaluate factors associated with survival to hospital discharge. After adjusting for confounders, HFOV before ECMO cannulation was an independent predictor for survival to hospital discharge (OR 28.44, *p* < 0.001). Other predictors of survival were the absence of hemorrhagic (OR 3.51, *p* 0.031) and renal (OR 3.50, *p* 0.027) complications during ECMO support. Table 3 summarizes the logistic regression analysis final model after backward selection.

**Table 3:** Fully adjusted multivariable logistic regression to ascertain factors associated with survival to hospital discharge.

Predictor	OR (95% CI)	*p*-value
LOS (days)	1.03 (1.01, 1.04)	**<0.001**
Ventilator type (HFOV vs Conventional)	28.44 (3.52, 229.58)	**<0.001**
Ventilator type (Other vs Conventional)	0.08 (0, 1.5)	**<0.001**
Absence of hemorrhagic complication	3.51 (1.12, 11.05)	**0.031**
Absence of renal complication	3.50 (1.15, 10.63)	**0.027**

CI: confidence interval, HFOV: High-frequency oscillatory ventilation, LOS: length of stay, OR: odds ratio.

## Discussion

This study is the first to date to uniquely characterize predictors of survival to hospital discharge in pediatric cardiac patients with PH requiring ECMO. In our study, HFOV before ECMO cannulation was an independent predictor for improved survival to hospital discharge in this cohort. The absence of renal and hemorrhagic complications were also independent predictors of survival in this population. This finding is supported by prior research that examined the impact of ECMO complications on patient mortality [15, 16].

Pediatric patients with heart disease are at heightened risk for acute PH due to several physiologic factors inherent to their disease process such as increased pulmonary pressures associated with a systemic to pulmonary shunt or elevated downstream pressures (such as left atrial hypertension), formation of arteriovenous malformations, development of veno-occlusive disease or association with bronchopulmonary abnormalities, etc. [3, 4]. Genetic abnormalities (such as Trisomy 21) commonly associated with CHD also independently place these patients at increased risk for PH [17, 18]. Furthermore, while traditionally hemoptysis, pulmonary infiltrates, and anemia are seen in patients with PH, these are often non-existent or attributed to other causes in infants and children with CHD complicating an early PH diagnosis [3, 5].

HFOV offers the theoretical benefit of minimizing ventilator-associated lung injury but data supporting positive outcomes have varied in previous research [19]. When employed in patients with acute PH, HFOV offers the advantage of high mean airway pressures that could help tamponade ongoing bleeding [14]. HFOV however is traditionally used with hesitancy in pediatric patients with heart disease due to the potential effects of increased intrathoracic pressure in reducing pulmonary venous return and/or increasing right ventricular afterload, thereby decreasing overall cardiac output [14]. However, some studies did not demonstrate hemodynamic deterioration using HFOV in non-operated and postoperative pediatric heart disease patients [14, 20]. Our study indicates that the use of HFOV in pediatric patients with heart disease and acute PH is a feasible option that may have contributed to improvement in survival. This may be attributable to the control of alveolar hemorrhage and subsequent improvement in lung compliance and gas exchange leading to better outcomes. Patients in the HFOV group were likely predisposed to respiratory conditions with severe oxygenation deficits. Variations in disease characteristics and comorbidities between ventilation groups, not captured by the ELSO registry, may have also influenced differences in survival.The use of ECMO for severe respiratory failure due to PH was historically discouraged given the need for systemic anticoagulation. However, several pediatric case studies have demonstrated that ECMO is feasible to manage life-threatening PH refractory to conventional therapy and allows time for diagnosis-directed therapies [7–13]. The observed survival benefit identified in this study and the paucity of prior research examining this association proffers an excellent opportunity to prospectively evaluate ventilatory strategies in pediatric cardiac patients with PH before ECMO cannulation.

The primary strength of this study is that it represents the largest and first report of pediatric cardiac patients with PH on ECMO including international multicenter data. Nonetheless, we acknowledge several limitations. It is a retrospective database review that depends on accurate data reporting from multiple ECMO centers worldwide. Center variation in the use of ECMO could not be accounted for. Additionally, while a statistically significant survival benefit was identified with the utilization of HFOV before ECMO cannulation in these patients, the power of this association is limited by the small number of patients who utilized HFOV compared to other ventilatory strategies. Furthermore, underlying differences in disease characteristics between patients on conventional modes vs HFOV, which are not fully captured in the ELSO registry, may have also contributed to these findings. Finally, we were not able to adjust for unreported factors associated with worse outcomes such as severity of illness scores, congenital heart surgery procedural scores, and unrecorded comorbidities.

In conclusion, in pediatric cardiac patients with acute pulmonary hemorrhage, the use of HFOV before ECMO cannulation is independently associated with improved survival. A prospective evaluation of mechanical ventilation practices preceding ECMO may enhance outcomes in this medically complex population.

## Data Availability

The data are available from the corresponding author on request (with permission from the ELSO).
